# EXAMINING THE IMPACT OF EXTREME HEAT TEMPERATURES IN FOOD INSECURITY AMONG LOW INCOME OLDER ADULTS

**DOI:** 10.1093/geroni/igae098.2320

**Published:** 2024-12-31

**Authors:** Jennifer Crook, Dahee Kim, Rui Xie, Christopher Emrich, Timothy Hawthorne, YunYing Zhong, James Brightman, Ladda Thiamwong

**Affiliations:** University of Central Florida, Orlando, Florida, United States; University of Central Florida, Orlando, Florida, United States; University of Central Florida, Orlando, Florida, United States; University of Central Florida, Orlando, Florida, United States; University of Central Florida, Orlando, Florida, United States; University of Central Florida, Orlando, Florida, United States; University of Central Florida, Orlando, Florida, United States; University of Central Florida, Orlando, Florida, United States

## Abstract

Extreme heat significantly impacts health outcomes in low-income older adults (LOAs), though a better understanding of the mediating effects in food insecurity (FI) has not been fully appreciated. Food insecurity is the lack of or inability to obtain adequate nutrition and the added burden of extreme weather overwhelms already tenuous household environments, especially in LOAs with limited resources. Through onsite interviews, our team sought to uncover the impact of extreme heat in LOAs (n=45) from low-income communities in Central Florida. We assessed FI with the U.S. Food Security Survey Module (US FSSM) 10-item questionnaire. Participants reported either food secure (FS) (n=32) or FI (n=13) status. Statistically significant impacts of extreme heat or hot weather were identified in health and day-to-day activities (x2[df=1] = 5.27, p = 0.02) with mean rank scores of FS 18.6 and FI 27.9 and sleep (x2[df=1] = 6.25, p = 0.01), with mean rank scores of FS 16.2 and FI 25.6, though none were observed for food or hydration or connection with friends. Significant barriers to community resource utilization included transportation (x2[df=1] = 9.99, p = 0.002) and distance from home to the center (x2[df=1] = 4.54, p = 0.03), but not time, health issues, or knowledge about services provided. No significant findings were detected in reduced physical activity, staying inside, visiting a cool place, or staying hydrated. Severely hot weather temperatures facilitate increased barriers in LOAs living with FI. Future efforts should consider FI when designing interventions targeted towards LOAs living in hot climate conditions.

